# Bacterial functional traits: a key driver of soil organic carbon dynamics during reductive soil disinfestation

**DOI:** 10.3389/fmicb.2026.1784827

**Published:** 2026-04-22

**Authors:** Risheng Xu, Haijiao Liu, Juan Liu, Yafei Chen, Ruiwei Ran, Lina Gao, Weizhen Zhao, Xing Chen, Tengfei Ma

**Affiliations:** 1 Peanut Research Institute, Henan Academy of Agricultural Sciences, Zhengzhou, China; 2 Sanming University, Sanming, China

**Keywords:** bacterial function traits, microbial community, necromass carbon, reductive soil disinfestation, soil organic carbon

## Abstract

Reductive soil disinfestation (RSD) significantly enhanced SOC content. However, its microbial mechanisms mediated through changes in microbial necromass carbon, particularly under different organic amendments, remain poorly understood. Here, we evaluated the effects of RSD combined with two types of organic amendments (wheat *vs*. soybean straw) on soil chemical properties, SOC fractions, microbial community composition and diversity, 16S rRNA gene operon (*rrn*) copy number, co-occurrence networks, and functional profiles. The results showed that microbial necromass carbon increased markedly after RSD treatment and accounted for up to 75% of the total SOC. Compared to wheat straw, soybean straw amendment resulted in higher relative abundances of Firmicutes (47.70%), Halobacterota (506.99%), and Euryarchaeota (1671.36%), intensified anaerobic conditions during RSD, improved suppression of aerobic fungi, and preferentially promoted the accumulation of fungal necromass carbon (33.25%). Conversely, the wheat straw treatment induced moderate anaerobic conditions, less pronounced than with soybean straw but stronger than the control, while sustaining higher bacterial abundance and promoting more robust bacterial succession, thereby preferentially leading to accumulation of bacterial necromass carbon (141.74%). These findings underscore the important role of bacteria functional traits in mediating carbon sequestration under anaerobic straw incorporation and provide new insights for optimizing organic amendment strategies in RSD practices to enhance soil carbon sequestration efficiently.

## 1 Introduction

Continuous cropping obstacles occur in multiple dimensions, affecting soil health, microbial communities, and crop performance (Teng et al., 2015; Zhou et al., 2023). Among them, the most significant impact is the degradation of soil fertility, which severely undermines soil quality, lowers crop productivity, and jeopardizes agroecosystem sustainability (Teng et al., 2015). To mitigate continuous cropping-induced soil degradation, common agricultural practices focus on replenishing soil organic carbon (SOC) through direct application of field residues. Among these, straw incorporation and straw-derived reductive soil disinfestation (RSD) have emerged as particularly effective strategies for enhancing SOC content (Chen et al., 2023a). However, existing research on RSD has largely neglected the central role of microbial necromass carbon (MNC) in SOC formation (Chen et al., 2023b). Currently, RSD is evolving as a green alternative to chemical fumigation, moving towards diversified carbon sources and refined operational protocols. However, this technology still faces several challenges, including significant variability in effectiveness due to carbon sources and insufficient understanding of its underlying soil microbial mechanisms. Existing research on RSD has largely neglected the central role of microbial necromass carbon (MNC) in SOC formation (Chen et al., 2023b). Elucidating the mechanisms through which MNC contributes to SOC accumulation, and the relationships between distinct MNC components and specific SOC pools would address critical gaps in current RSD research and help advance its optimization and application.

Soil microorganisms play a central role in governing SOC dynamics, as their growth and activities mediate the soil C dynamics: formation, transformation, stabilization, and decomposition (Liang et al., 2017; Chen et al., 2023a). In particular, the formation and stabilization of SOC constitute a complex process driven by the microbial transformation of plant residues through two primary pathways: (i) *ex vivo* modification, primarily involving exoenzymatic decomposition and catabolism, and (ii) *in vivo* anabolic processes, encompassing microbe uptake, biosynthesis, growth, and death. Microbial necromass, originating from in vivo anabolic processes, constitutes a critical component of the SOC stock (Liang, 2020; Zhang et al., 2024). Given its contribution of ~51% to cropland SOC, MNC is established as a primary driver of slow-cycling SOC pool formation (Wang B. et al., 2021). Under RSD treatment, application of labile carbon sources (e.g., straw, green manure, molasses) followed by waterlogging and impermeable film coverage (~21 days) establishes highly reductive microenvironments (Momma et al., 2013; Ueki et al., 2018). Compared to aerobic agricultural systems, RSD induces severely anaerobic conditions and substantial microbial mortality, which likely fundamentally alters the sources (bacterial versus fungal), turnover rates, and stabilization pathways of microbial necromass carbon, thereby more strongly amplifying necromass-derived carbon pools (Lopes et al., 2022). Previous study demonstrated that RSD induced a net SOC increase of 1.26 g C kg^−1^, resulting from the balance between newly formed SOC (+1.82 g C kg^−1^) and mineralization losses of native SOC (−0.56 g C kg^−1^) (Chen et al., 2023b). Consequently, RSD represents a dual-functional strategy for rehabilitating degraded soils while concurrently enhancing carbon sequestration. Nevertheless, the mechanisms governing microbe-mediated SOC stabilization remain inadequately resolved.

Microbial functional traits-including biophysical properties, cellular stoichiometry, and life-history strategies-fundamentally regulate SOC cycling and MNC accumulation (Yang et al., 2024; Sun and Han, 2025). For instance, copiotrophic microbes rapidly assimilate monomeric substrates into necromass that stabilizes SOC. Conversely, in oligotrophic environments, metabolic allocation toward exoenzyme synthesis for nutrient mining stoichiometrically compromises growth efficiency, thereby suppressing biomass-derived carbon accrual (Tang et al., 2024). Recently, the 16S rRNA gene operon (*rrn*) copy number serves as a robust proxy for different microbial nutrient use efficiency and growth rates. This trait elucidates microbial adaptations across environmental gradients: faster-growing taxa with higher *rrn* copy numbers invest heavily in ribosome synthesis to support rapid growth, often at the expense of metabolic enzyme production and nutrient use efficiency; conversely, under nutrient-limited conditions, oligotrophic organisms with lower *rrn* copy numbers allocate more energy toward metabolic enzymes (He et al., 2023; Tang et al., 2024). Critically, observed community-level correlations between *rrn* copy number and nutrient cycling underscore its utility as a functional indicator of SOC availability the abundance of genes involved in carbon and nutrient cycling underscore its utility as a functional indicator of SOC availability (Sun and Han, 2025). Following organic amendment and plastic-mulched in RSD, the soil transitions from resource-limited to copiotrophic conditions, triggering rapid proliferation of r-strategists (predominantly anaerobic bacteria) that mean rapidly assimilate monomeric substrates into bacterial necromass. Meanwhile, substantial aerobic fungal populations undergo significant mortality, further increasing fungal necromass accumulation (Lopes et al., 2022; Chen et al., 2023a). However, it remains uncertain which MNC sequestration pathway predominates in different organic amended RSD systems, nor are the underlying mechanisms understood.

In this study, we investigated the relationships between distinct functional traits, focusing on the *rrn* copy number as a key indicator of bacteria under different organic amendments applied to RSD, and their associations with MNC components. This exploration aimed to elucidate the mechanisms underlying microbe-mediated SOC accumulation of RSD with two types of organic amendments. We hypothesized that: (1) The C:N ratio of the organic amendment critically shapes the accumulation pathways of MNC: materials with lower C:N ratios (e.g., soybean straw) can rapidly stimulate oxygen consumption, thereby promoting stronger anaerobic conditions that enhance the suppression of aerobic fungi and favor the accumulation of fungal-derived necromass carbon. In contrast, materials with higher C:N ratios (e.g., wheat straw) degrade more slowly due to their higher cellulose content, leading to less intensive anaerobic conditions and sustaining a larger and more active cellulose-degrading bacterial community, which in turn may favor the accumulation of bacterial necromass carbon; and (2) in both pathways, the proliferation of anaerobic bacteria stimulated by RSD may play a pivotal role in accelerating soil SOC accumulation via microbial necromass carbon. This study provides a theoretical microbial foundation for soil SOC accumulation following RSD treatment, as well as for optimizing the RSD technology and selecting relevant key parameters.

## 2 Materials and methods

### 2.1 Soil samples for the experiment

Soil samples were collected from a field under long-term peanut monoculture in the vicinity of decommissioned mines in Mianxian Town (33°15′35″N, 106°37′32″E), Hanzhong City, Shaanxi Province, China. The soil, classified as Luvisols (FAO, 2006), is characterized by low fertility and a high incidence of soil-borne diseases. The local climate features a mean annual temperature of 14.2 °C and an annual precipitation of 841 mm. Prior to sampling, the site had been used for peanut cultivation during the previous growing season. The SOC content was 5.86 g kg^−1^. Other properties included: pH 5.81, NH_4_^+^–N 1.60 mg kg^−1^, NO_3_^−^–N 205.23 mg kg^−1^ (Xu et al., 2025a). The collected samples were sieved, homogenized, and subdivided for subsequent experimental use.

### 2.2 Experimental treatments

Six treatments were established: (1) Control soil (CK); (2) Water flooding without straw (WF); (3) Water flooding with low-dose (1.5 g kg^−1^, equivalent to field application rate of 3.2 Mg hm^−2^) soybean straw (LSD); (4) Water flooding with high-dose (7 g kg^−1^, equivalent to field application rate of 15 Mg hm^−2^) soybean straw (HSD); (5) Water flooding with low-dose (1.5 g kg^−1^) wheat straw (LWD); (6) Water flooding with high-dose (7 g kg^−1^) wheat straw (HWD). Wheat and soybean straw were obtained at maturity from local fields, ground, and sieved to a particle size of <2 mm. The wheat straw contained 331.25 g kg^−1^ C and 4.13 g kg^−1^ N, while the soybean straw contained 465.47 g C kg^−1^ and 26.32 g N kg^−1^. Each foam box (212 mm length × 115 mm width × 140 mm height × 15 mm thickness) was filled with 500 g soil, either untreated or amended with the specified straw additives. The experiment was arranged in a randomized complete block design with three replicates per treatment (6 treatments × 3 replicates = 18 boxes). The CK group was maintained at 70% field water capacity through periodic irrigation for 30 days. All other treatments (WF, LSD, HSD, LWD, HWD) were subjected to anaerobic conditions for 30 days by maintaining a 3 cm water layer above the soil surface and sealing with transparent polyolefin film (0.08 mm thick; Luchen Plastics Co., Weifang, China). All boxes were incubated in a greenhouse at 25–35 °C.

### 2.3 Sample collection and analysis

After the incubation period, the plastic film was removed, and three randomly sampled soil cores from each replicate were combined, homogenized, and split into two portions. One subsample was freeze-dried and stored at −80 °C for subsequent phospholipid fatty acid (PLFA), amino sugar, and high-throughput sequencing analyses. The other portion was air-dried and sieved (0.15 mm) for soil chemical properties and SOC fractions determination.

Soil chemical properties-soil organic carbon (SOC), soil redox potential (Eh), electrical conductivity (EC), pH, ammonium nitrogen (NH₄^+^–N), nitrate nitrogen (NO₃^−^–N), and available phosphorus (AP) were measured following the methods outlined in Xu et al. (2025b).

Amino sugars were extracted by hydrolyzing soil samples with 6 M HCl at 105 °C for 8 h. The hydrolyzate was filtered, neutralized (pH 6.6–6.8), and centrifuged (4,000 r/min, 10 min). The supernatant was lyophilized, and the residue was re-dissolved in methanol for amino sugar recovery. After adding myo-inositol as an internal standard, amino sugars were derivatized into aldononitrile acetates following Shennan et al. (2017). Derivatized samples were separated using an HP-5 fused silica column (25 m × 0.32 mm × 0.25 μm) on an Agilent 7890B gas chromatograph equipped with a flame ionization detector. Total amino sugar content was calculated as the sum of glucosamine, galactosamine, mannosamine, and muramic acid (Eudy et al., 1985). Bacterial microbial residue carbon (MRC) was estimated by multiplying muramic acid concentration by 45. Fungal MRC was derived by subtracting bacterial muramic acid from total glucosamine, based on a assumed molar ratio of glucosamine to muramic acid of 2:1 in bacteria (Amelung et al., 2002; Engelking et al., 2007). Total MRC was defined as the sum of fungal and bacterial MRC.

### 2.4 Genomic DNA extraction and high-throughput sequencing

Genomic DNA was extracted from 0.5 g of soil per replicate using the FastDNA™ Spin Kit for Soil (MP Biomedicals, Solon, OH, USA) according to the manufacturer’s instructions. The concentration and purity of the DNA were assessed with a NanoDrop ND-1000 spectrophotometer (Thermo Fisher Scientific, Wilmington, DE, USA). The V4 region of the bacterial 16S rRNA gene was amplified with primers 515F (5′-GTGCCAGCMGCCGCGG-3′) and 907R (5′-CCGTCAATTCCTTTGAGTTT-3′) (Xu et al., 2025b). The fungal internal transcribed spacer (ITS) region was amplified using primers ITS1F (5′-CTTGGTCATTTAGAGGAAGTAA-3′) and ITS2R (5′-GCTGCGTTCTTCATCGATGC-3′) (Xu et al., 2025b). PCR was performed using a GeneAmp® 9,700 thermal cycler (Applied Biosystems, Foster City, CA, USA) under cycling conditions adapted from previous work (Xu et al., 2025b).

Amplified products were purified with the AxyPrep™ DNA Gel Extraction Kit (Axygen Biosciences, Union City, CA, USA) and quantified using the QuantiFluor™-ST Fluorometer (Promega, Madison, WI, USA). Sequencing libraries were constructed, followed by cluster generation and 250-bp paired-end sequencing on an Illumina MiSeq platform (Illumina, San Diego, CA, USA), which was conducted by Genesky Biotechnologies Inc. (Shanghai, China) (Xu et al., 2025b).

Further details regarding bioinformatic processing, sequence analysis, functional prediction, and co-occurrence network construction are provided in the Supplementary methods.

### 2.5 Statistical analysis

Differences in soil and microbial properties among treatments were assessed using one-way ANOVA, followed by Tukey HSD’s multiple comparison test at significance levels of *p* < 0.05. Where the assumptions of normality or homogeneity of variances were violated, Welch’s *t*-test (which does not assume equal variances) was applied, and *p*-values were corrected using the Benjamini–Hochberg method. Statistically significant differences are indicated with asterisks in the corresponding figures and tables. All statistical computations were conducted using SPSS 18.0 (SPSS Inc., Chicago, IL, USA). Microbial community composition differences were examined via principal coordinates analysis (PCoA) and visualized with Canoco 5.0 (Microcomputer Power, Ithaca, NY, USA). Heatmaps generated with the R package heatmap3 were used to display spearman’s correlations between microbial communities and soil properties and SOC fractions. To identify microbial biomarkers from phylum to genus level, linear discriminant analysis effect size (LEfSe) was performed,^1^ with a linear discriminant analysis (LDA) score threshold set at 2.0.

## 3 Results

### 3.1 SOC fractions

**Figure 1:**
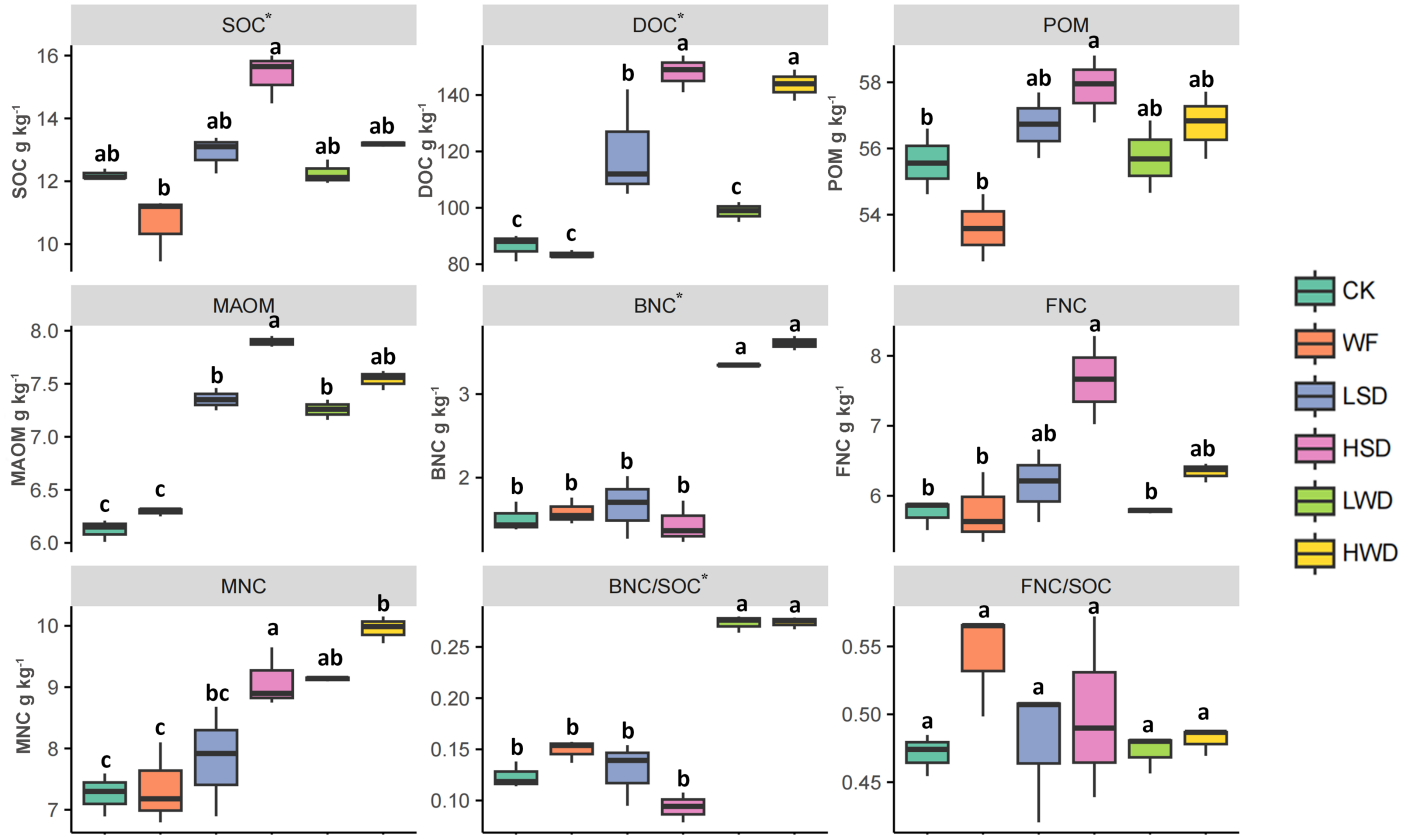
Effects of anaerobic soil disinfestation on soil organic carbon (SOC; g kg^−1^), dissolved organic C (DOC; g kg^−1^), particulate organic matter (POM; g kg^−1^), and mineral-associated organic matter (MAOM; g kg^−1^); bacterial necromass carbon (BNC), fungal necromass carbon (FNC), and microbial necromass carbon (MNC). The different letters indicate significant differences between the treatments (*p* < 0.05). Control soil (CK); water flooding without straw (WF); water flooding with low-dose (1.5 g kg^−1^) soybean straw (LSD); water flooding with high-dose (7 g kg^−1^) soybean straw (HSD); water flooding with low-dose (1.5 g kg^−1^) wheat straw (LWD); water flooding with high-dose (7 g kg^−1^) wheat straw (HWD).

The results demonstrated that the different organic material RSDs led to varying increases in the contents of SOC fractions compared to the control treatment (Figure 1). Specifically, the HSD treatment elevated SOC, DOC, and MAOM contents by 26.23, 71.30, and 29.51%, respectively. Similarly, the HWD treatment significantly raised DOC and MAOM contents by 66.35 and 23.14% (*p* < 0.05). All RSD treatments with different organic materials exhibited a clear dose–response relationship, with higher straw application rates leading to greater accumulation of SOC fractions. BNC or BNC/SOC levels increased significantly following wheat straw addition, with the LWD and HWD treatments showing significantly higher values than those in the CK, WF, LSD, and HSD treatments. Obviously, wheat straw addition significantly increased MNC/SOC to 74.59 and 75.49%, respectively (*p* < 0.05) (Supplementary Figure S1). Conversely, only the HSD treatment led to a marked increase in FNC concentration compared to the CK and WF treatments.

### 3.2 Microbial community composition

**Figure 2:**
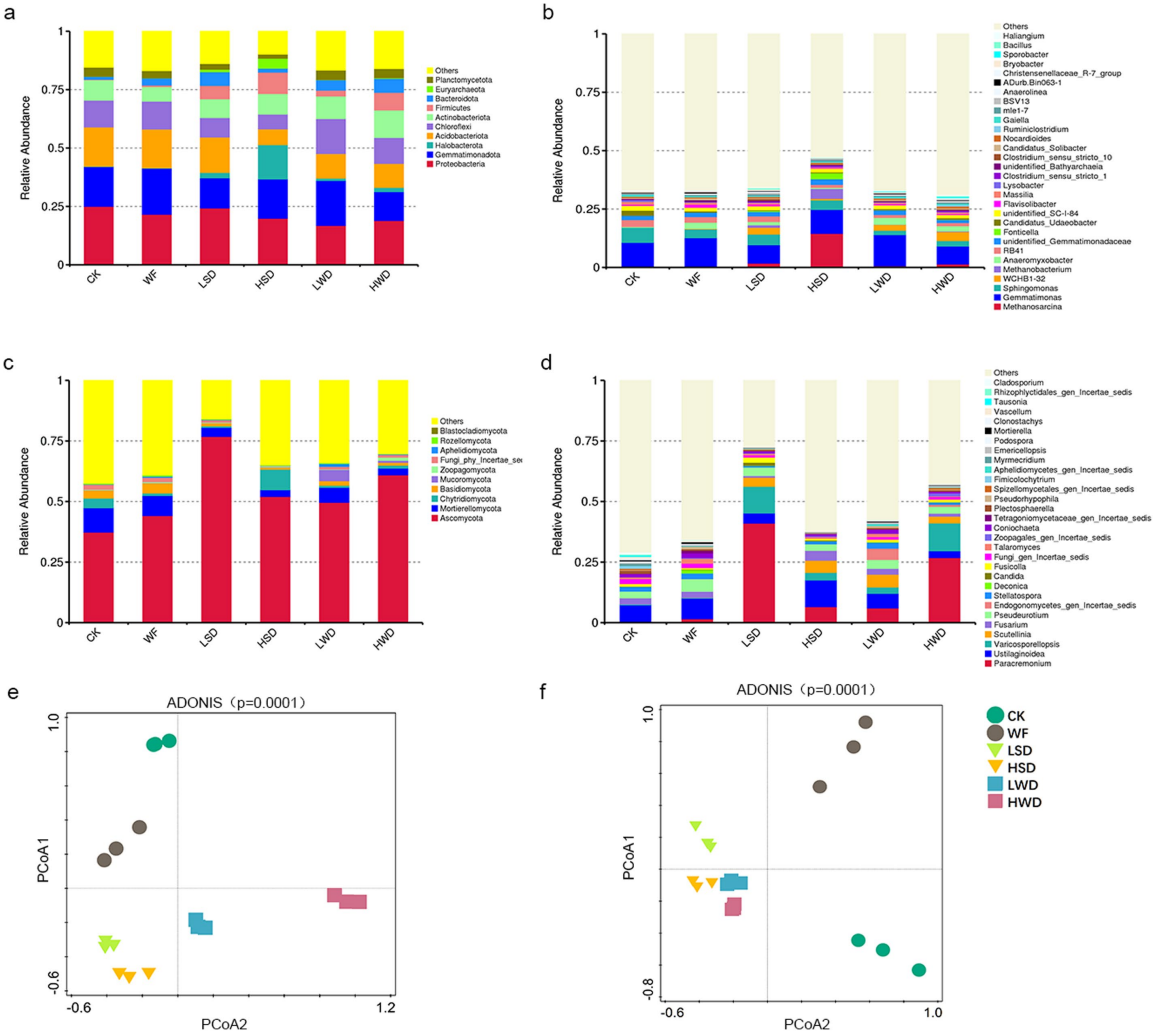
Community composition of soil bacteria and fungi. Relative abundance of soil bacteria [**(a)** Phylum level; **(b)** genus level] and fungi [**(c)** phylum level; **(d)** genus level]. Groups with <0.01 relative abundance were merged into the “others” group. Principal coordinate analysis (PCoA) of bacterial **(e)** and fungal **(f)** communities at the ASV level. Treatments, values, and different letters were defined in Figure 1.

For bacterial communities in soybean straw addition treatments, LSD treatment decreased the proportions of Chloroflexi, Gemmatimonadota, and Planctomycetota but increased those of Firmicutes and Bacteroidota compared to those under CK and WF treatments (Figure 2a). Specifically, a shape increase of Firmicutes (26.13%), archaea Halobacterota (13.39%) and Euryarchaeota (100.61%) was observed in LSD compared to those under CK. Moreover, HSD treatment further increased the proportions of bacterial phyla Firmicutes (by 42.53%), archaea phyla Halobacterota (by 92.01%) and Euryarchaeota (by 406.87%) based on the levels of CK. Correspondingly, at the genus level, the relative abundance of archaea genera such as *Methanosarcina* and *Methanobacterium* markerly increased in the LSD and HSD treatments (Figure 2b). In wheat straw addition treatments, LWD and HWD treatments decreased the proportions of Gemmatimonadota but increased those of bacterial phyla Firmicutes (by 22.92%), archaea phyla Halobacterota (by 784.66%) compared to those under CK and WF treatments (Figure 2a). The results of PCoA showed that samples from all treatments were separated from each other (Figure 2e).

For fungi, both soybean straw and wheat straw additions exhibited similar community composition among different treatments, with variations in the relative abundance of specific fungal species in different treatments (Figures 2c,d). The fungal community compositions of PCoA plot also showed that samples from all straw additions clustered together and were separated from the CK and WF treatments (Figure 2f). The adonis analyses indicated that straw addition (*p* < 0.01) significantly affected fungal community compositions (Figure 2). Specifically, in the straw addition treatments, the relative abundance of the phylum Ascomycota were higher in the LSD and HWD treatments (Figure 2c). More pronounced differences were observed at the genus level in phylum Ascomycota (Figure 2d), for example, the relative abundance of abundant genera, such as *Paracremonium* and Var*icosporellopsis* were markedly increased in the LSD and HWD treatments compared to that in CK and WF treatments.

### 3.3 Predicted functional properties

**Figure 3:**
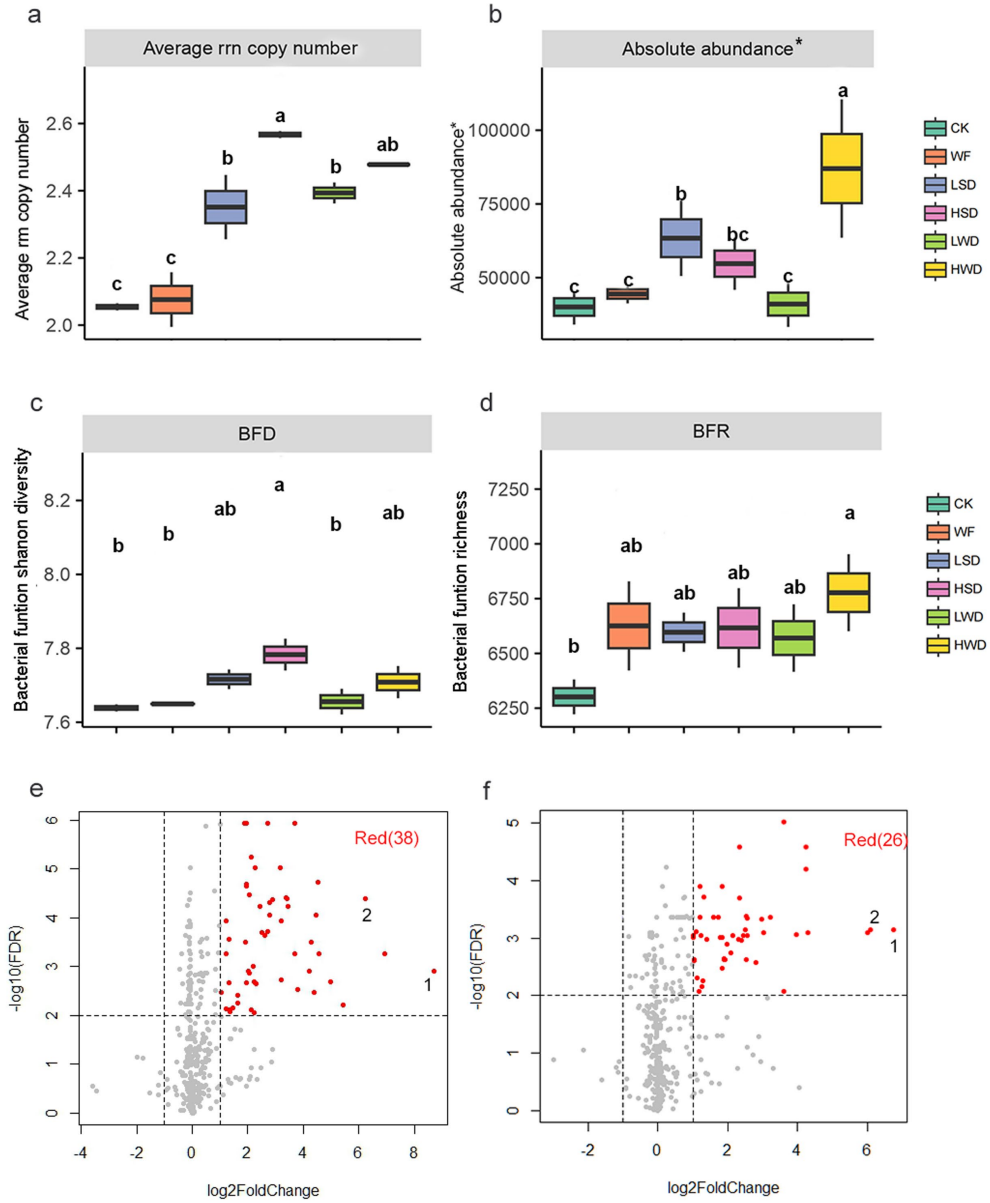
Effects of anaerobic soil disinfestation on the bacterial community-level *rrn* copy numbers **(a)**, bacterial absolute abundance **(b)**, bacterial function Shannon diversity **(c)**, bacterial function richness **(d)**. Volcano plots showing significantly enriched metabolic pathways in soybean straw **(e)** and wheat straw **(f)** treatments. Red points and numbers indicated significantly higher abundances of metabolic pathway in treatments compared with the control (*p* < 0.05). 1: Superpathway of methanogenesis; 2: Chitin derivatives degradation.

The bacterial community-level *rrn* copy number in LSD, HSD, LWD and HWD was significantly (*p* < 0.05) higher than that in CK and WF, with increased by 13.85, 24.30, 15.89, and 19.98%, respectively (Figure 3a). However, only a marked increase in absolute ahundance of bacteria was evident in the HWD soil compared with that in the CK and WF soil (Figure 3b).

It was showed that different organic material RSDs led to the rise in the BFD (bacterial funtion shanon diversity) and BFR (bacterial funtion richness) to different extent when compared to control treatment (Figures 3c,d). Specifically, HSD treatments significantly increased BFD, with 1.97% (*p* < 0.05); while HWD increased BFR, with 7.55% but it was not reach significant. In addition, 38 and 26 bacterial groups were significantly differentially enriched in soybean straw and wheat straw addition treatments, respectively (Figures 3e,f). Furthermore, the functional categories of bacterial enrichment were broadly similar between the two straw types; however, enrichment was significantly higher in soybean straw compared to wheat straw additions. For instance, superpathway of methanogenesis increased by 8.70 log fold change in soybean straw, compared to 6.76 log fold change in wheat straw.

### 3.4 LEfSe analysis of microbial communities and spearman’s correlations

**Figure 4:**
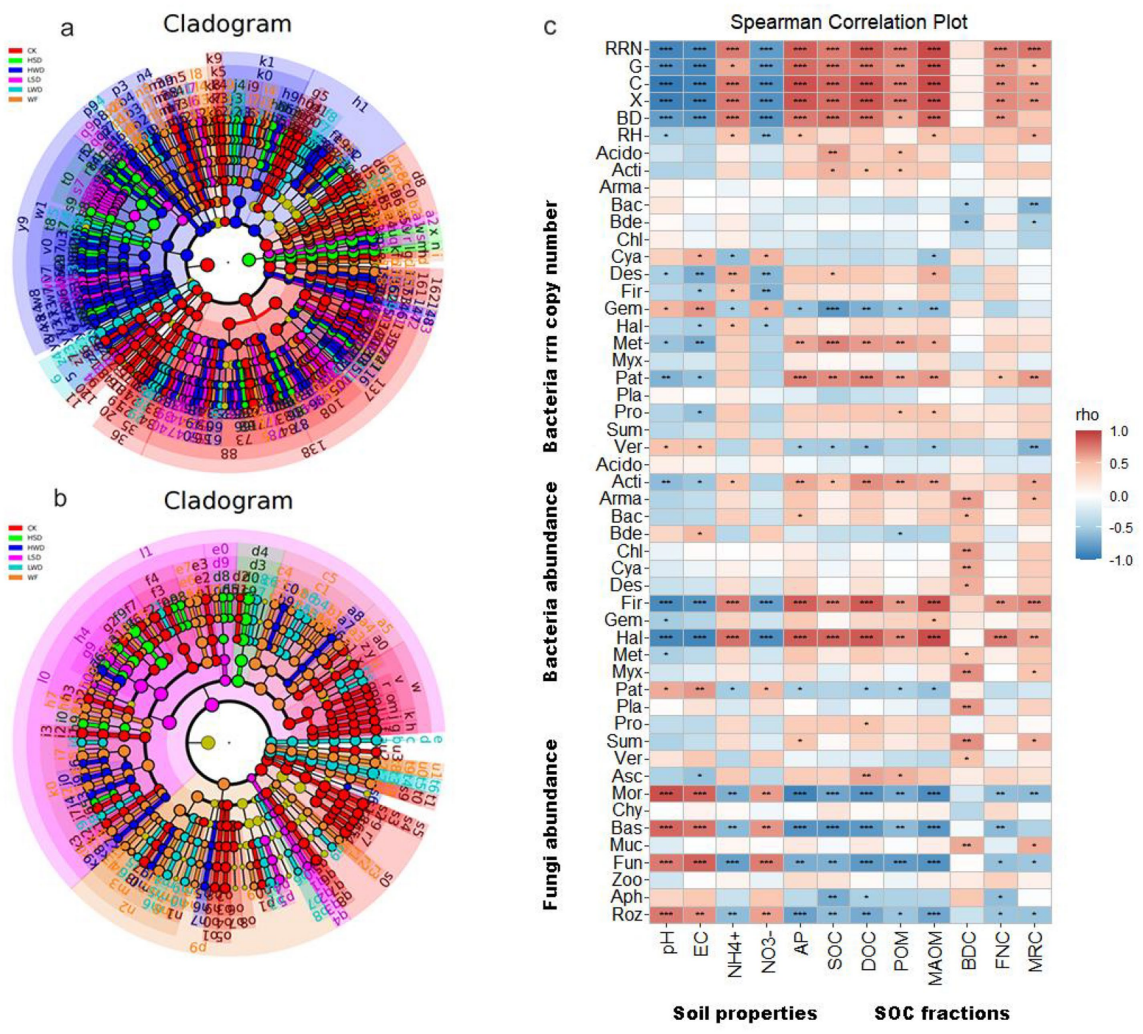
Linear discriminant analysis effect size (LEfSe) analysis of bacterial **(a)** and fungal **(b)** taxa significantly enriched across all treatments under different organic amendments. Spearman correlations **(c)** represent the interactions between microbial communities and soil properties and SOC fractions, asterisks indicate significant differences (^***^p < 0.001, ^**^p < 0.01, ^*^p < 0.05).

LEfSe analysis revealed significant variations in the bacterial and fungal communities in response to the addition of soybean straw and wheat straw (Figures 4a,b). Specifically, the numbers of significantly differentially enriched bacterial groups were 159, 84, 102, 66, 56, and 172 under the CK, WF, LSD, HSD, LWD, and HWD treatments, respectively. In contrast, the corresponding numbers for fungal groups were 109, 91, 19, 26, 52, and 29, respectively. Notably, the number of responsive taxa was greater in bacterial communities than in fungal communities, particularly in straw-amended soils.

To further explore the associations between microbial communities and soil properties and SOC fractions, we calculated the Spearman’s rank correlation coefficients (Figure 4c). *rrn*, β-1,4-glucosidase activity, Cellobiohydrolase activity, β-1,4-xylosidase activity, and BFD were negatively correlated with pH, EC, NO_3_^−^-N and positively associated with NH_4_^+^-N, AP, SOC, DOC, POM, MAOM, and FNC. For *rrn* copy number, various bacterial phyla such as Desulfobacterota, Gemmatimonadota, Methylomirabilota, and Halobacterota were more strongly influenced by soil properties and SOC fractions. However, the abundance of bacterial phyla Firmicutes and Halobacterota was negatively correlated with pH, EC, and NO_3_^−^–N, but positively associated with NH_4_^+^–N, AP, SOC, DOC, POM, MAOM, and FNC. Conversely, the abundance of fungal phyla Mortierellomycota, Basidiomycota, Fungi_phy_Incertae_sedis, and Rozellomycota showed strong inverse correlations with these same indicators.

### 3.5 Microbial network properties

**Figure 5:**
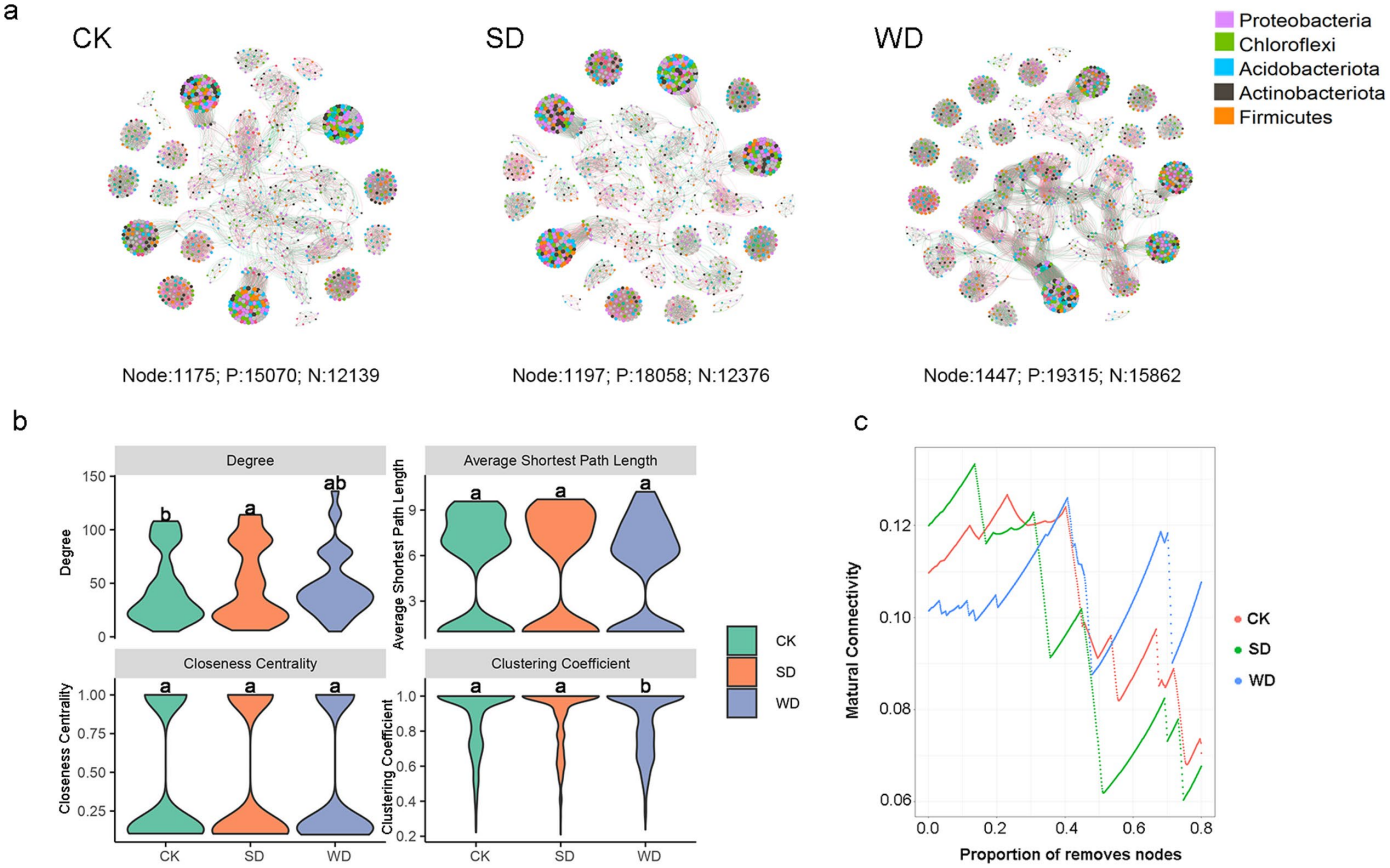
Co-occurrence networks of soil bacteria **(a)**. The size of each node is proportional to the number of connections (degree). The red edges indicate positive interactions between two nodes, while the green edges indicate negative interactions. The nodes (at the genus level) are colored according to bacteria phyla. Node, P, and N in networks represent nodes, positive interactions, and negative interactions of edges, respectively. The degree, average shortest path length, closeness centrality, and clustering coefficient values of the nodes and stability of the bacterial network under different organic amendments **(b)**. Control and water flooding (CK); soybean straw (SD); wheat straw (WD) **(c)**.

To identify the effect of abandonment on potential bacteria and bacteria interactions, bacterial co-occurrence networks were constructed between soybean (SD) and wheat (WD) straw additions (Figure 5). The results showed that SD resulted in clear increases in network positive relationships, furthermore, WD resulted in clear increases in network nodes, positive and negative relationships, with 23.15, 28.17, and 30.67%, respectively.

It was showed that different organic material RSDs led to the rise in the degree, average shortest path length, closeness centrality, and clustering coefficient values to different extent when compared to control treatment. Moreover, WD treatment further increased the these same indicators based on the levels of LSD (Figure 5b).

To determine the effect of abandonment on the robustness of bacterial networks, a natural connectivity analysis between SD and WD soil was performed (Figure 5c). The results showed that SD soil had lower bacterial values than CK, ranging from 0.3–0.8, whereas the WD had higher bacterial values than CK, ranging from 0.5–0.8.

### 3.6 Linking straw properties, bacterial functions and enzyme activities to the SOC dynamics

**Figure 6:**
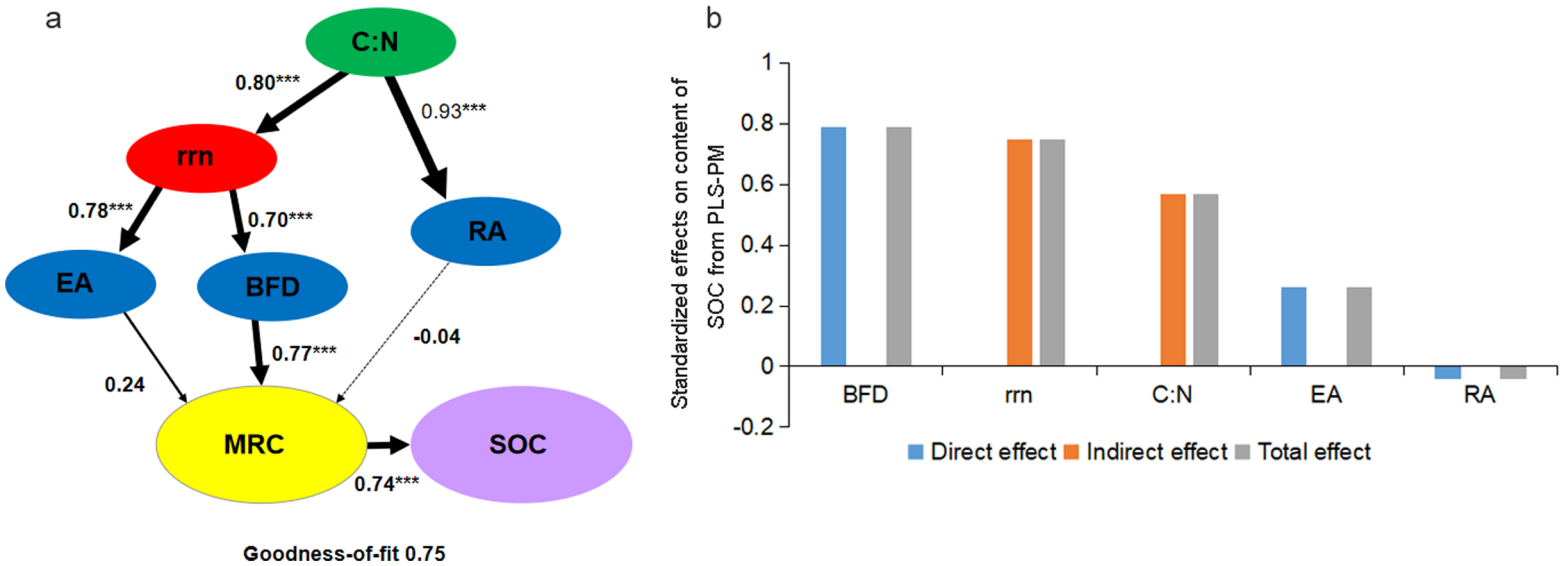
Partial least-squares-path modeling (PLS-PM) of straw amendment effects on soil organic carbon (SOC) and microbial residue carbon (MRC) **(a)** and corresponding standardized direct and indirect mean effects **(b)**. Larger path coefficients are shown as wider arrows, and solid and dotted line indicate positive and negative effects, respectively. Path coefficients and coefficients of determination (*R*^2^) were calculated after 999 bootstraps, and significance levels are indicated by ^*^(*p* < 0.05), ^**^(*p* < 0.01), and ^***^(*p* < 0.001). The goodness of fit (GoF) for the hyperthermophilic and conventional treatments was 0.75. *rrn*: bacterial community-level *rrn* copy numbers, EA: β-1,4-glucosidase activity, cellobiohydrolase activity, β-1,4-xylosidase activity, BFD: bacterial function Shannon diversity, RA: absolute abundance of bacterial phyla Firmicutes and archaea phyla Halobacterota, MRC: microbial residue carbon, SOC: soil organic carbon.

Partial least squares path modelling (PLS-PM) was used to assess the direct and indirect effects of straw addition on the MRC and SOC dynamics (Figure 6). The C:N ratios of soils weakly influenced the content of MRC via relative abundance of bacteria (path coefficient: 0.93, *p* < 0.001 and −0.04; *p* > 0.05). However, the indirect effect of C:N ratios on content of MRC via *rrn* and BFD was much stronger (path coefficients: 0.80, 0.70, and 0.77; *p* < 0.001).

## 4 Discussion

### 4.1 Impacts of different organic material RSDs on soil SOC fractions

Before RSD treatment, long-term continuous peanut cropping had led to severe soil degradation, with soil organic carbon (SOC) content as low as 5.86 g kg^−1^. However, RSD treatment effectively reversed this trend, significantly increasing the contents of SOC, DOC, POM, and MAOM, particularly with high dose soybean straw addition (Figure 1). This finding is consistent with the majority of domestic and international studies (Zhao et al., 2020; Chen et al., 2023a,b), indicating that straw incorporation measures such as RSD are a robust strategy for restoring SOC levels. Furthermore, our results highlight that MNC constitutes a substantial proportion of this increase (Supplementary Figure S1). This (75%) even was higher than previous research reporting that MNC accounts for 51.0% of soil carbon in croplands (Wang B. et al., 2021) and is recognized as a critical contributor to the formation of slow-cycling soil carbon pools.

Soil microbial necromass are considered primary regulators of soil carbon dynamics (Liang et al., 2017; Liang, 2020; Chen et al., 2023a). Our experimental results demonstrated that the accumulation of microbial necromass carbon varied depending on the type of organic amendment used in RSD: the application of soybean straw under flooded conditions primarily promoted the accumulation of FNC, whereas wheat straw with a higher C/N ratio mainly enhanced the accumulation of BNC. This divergence is likely closely related to the C/N ratio of the organic materials themselves. In fact, the C/N ratio is a key factor influencing crop straw decomposition (Zhao et al., 2020; Lopes et al., 2022). Wheat straw generally has a higher C/N ratio, and its microbial decomposition depends on native soil nitrogen, thereby slowing the decomposition rate (Xu et al., 2024). Conversely, soybean straw provides sufficient nitrogen to support rapid microbial degradation and oxygen consumption. Our results support this conclusion: compared to wheat straw, the application of soybean straw under RSD led to stronger anaerobic conditions, as indicated by a greater decrease in the oxidation–reduction potential (Supplementary Table S2), resulting in more effective suppression of aerobic fungi and consequently greater production of FNC (Liu et al., 2016). Furthermore, the relatively stronger effect of soybean straw under RSD likely promoted soil aggregate formation, increased iron oxide precipitation, and enhanced organic acid production, all of which could further protect newly formed organic carbon from degradation (Hu et al., 2024; Xu et al., 2024). On the other hand, although the decomposition of wheat straw with higher C/N ratios was limited by nitrogen scarcity, they better sustained the abundance and Shannon diversity of bacterial communities (Supplementary Tables S3, Table S4) compared to the soybean straw. PLFAs demonstrated that the wheat straw treatment groups retained a higher bacterial abundance compared to soybean straw treatment groups, particularly the anaerobic bacteria and Gram-positive bacteria (Supplementary Figure S2). Notably, *Pseudomonas*, known for its cellulose-degrading capabilities (Gao et al., 2016), was identified as the dominant genus in this treatment. Generally, higher microbial biomass concentration promotes the accumulation of more necromass (Wang B. et al., 2021), which explained the greater accumulation of BNC in the wheat straw treatment. Overall, the wheat straw treatments accumulated higher absolute amounts of MNC compared to soybean straw treatments, contributing up to 75% of the SOC content. This effect likely stems from the ability of wheat straw treatments to sustain a more diverse and abundant active bacterial community over a longer period, coupled with the accumulation of a certain amount of fungal necromass carbon, leading to a more integrated enhancement of microbial necromass carbon sequestration.

### 4.2 Influence of different organic material RSDs on bacterial and fungal community compositions and functions in soil

Soil microbes utilize plant-derived carbon for growth through biosynthesis and carbon from microbial necromass enters the soil carbon pool via the entombing effect (Liang et al., 2017; Liang, 2020). The microbial efficiency-matrix stabilization framework (Ji et al., 2025) suggests that high-quality plant residues such as soybean straw with higher nitrogen content are more readily decomposed and transformed by microbes, thereby contributing more effectively to stable soil organic matter formation, a conclusion supported by our findings (Figure 1). We further observed that the RSD treatment with soybean straw promote the accumulation of fungal necromass carbon. This effect is likely associated with the sterilization efficiency of RSD, the following two main reasons: First, high-quality straw residues led to a higher abundance of archaea, such as methanotrophs, while the abundance of strictly anaerobic microorganisms (such as certain archaea) was lower in low-quality straw treatments. This suggested that high-quality straw enhanced the anaerobic conditions during RSD, thereby improving the suppression of aerobic fungi. Additionally, organic acids produced by various anaerobic microorganisms further contribute to pathogen elimination, enhancing the sterilization effect in the high-quality straw treatment group (Liu et al., 2016).

Second, in terms of microbial function, the high-quality plant residues such as soybean straw treatment enriched anaerobic metabolic processes such as methane oxidation, with a quantified enrichment level of 8.70 log fold change. In contrast, the low-quality straw treatment showed a decrease in these functions, with an enrichment level of 6.76 log fold change (Figures 3e,f).

Microbial functional traits are specific characteristics that shape the roles of microorganisms in ecosystems, encompassing physiological, behavioral, and interactive attributes (Yang et al., 2024). Among these, the *rrn* serves as a valuable indicator of microbial growth rate and nutrient use efficiency (He et al., 2023; Zhang et al., 2024). This trait offers insight into microbial ecological strategies, from copiotrophic (r-strategist) tendencies in nutrient-rich environments to oligotrophic (K-strategist) traits under nutrient-limited conditions. Sun and Han (2025) reported a positive correlation between *rrn* copy number and microbial growth rate, and an inverse relationship with carbon use efficiency. Our *rrn* copy number results also showed that the high-quality straw treatment group had a higher average community *rrn* copy number, which was significantly greater than that in the low-quality straw group (Figure 3a). This suggested that an optimal carbon-to-nitrogen ratio may stimulate the growth of anaerobic r-strategists and enhancing the effectiveness of RSD. Meanwhile, a higher ribosomal content facilitates the translation of more functional proteins, thereby increasing community multifunctionality and indicating an enhanced capacity to utilize diverse organic substrates to sustain the rapid growth of r-strategists (He et al., 2025; Figure 3c). It should be noted that the *rrn* copy numbers inferred from phylogenetic approaches reflect the genomic potential of microbial taxa rather than their actual in-situ expression or activity levels in the current soil environment. Therefore, future studies should incorporate metagenomic or metatranscriptomic data to more directly validate the expression dynamics of related functional traits.

Analysis of fungal community structure via PCoA and functional trait prediction (FPLA) indicated that fungal communities converged across different straw treatments and were highly similar in composition except for a few abundant taxa (Liu et al., 2016). This suggests that RSD indiscriminately suppresses aerobic fungi. Moreover, functional annotation of fungal communities revealed a significant reduction in pathogenic fungi across all straw treatments, with no notable differences between the straw addition groups (Supplementary Figures S1, S3), further supporting this conclusion.

### 4.3 Effect of different organic material RSDs on bacterial network and stability

In the soybean addition group, a greater number of positive correlations were observed compared to the control. Since positive interactions within microbial networks are generally indicative of cooperation (Ma et al., 2020), the higher frequency of such interactions suggests that bacteria in the soybean addition group may collaborate more effectively to enhance functional performance. For instance, methane production, known to require syntrophic interactions among different microorganisms, is supported by modularity data (Supplementary Table S6), reinforcing this interpretation (Edwards et al., 2015). In contrast, the wheat addition group sustained a larger bacterial population (Figure 3b; Supplementary Figure S1), likely due to the persistence of harder-to-degrade straw residues that help maintain higher bacterial abundance over extended periods. Previous studies have shown that the decomposition of recalcitrant straw materials (e.g., cellulose and lignin) depends on complex interbacterial relationships and sustained microbial activity (Addo et al., 2021; Xu et al., 2024).

The different organic material RSDs also influenced the robustness of the bacterial network, indicating its potential impact on community stability. In this study, the soybean straw amendment enhanced the complexity of the bacterial network but diminished its stability and modularity. These findings align with those of Morriën et al. (2017) and Wu et al. (2024), who observed that reduced modularity and increased complexity were associated with decreased network stability. Specifically, greater complexity leads to more tightly coupled interactions among nodes, which can enhance overall connectivity in response to resource enrichment—such as increased organic carbon availability (Wu et al., 2024). Compared to the control, the network under soybean straw treatment showed little change in the proportion of negative correlations, but a marked increase in positive correlations. This pattern suggests an over-representation of positive cooperative interactions geared toward degrading soybean-derived carbon, which may contribute to structural over-centralization and reduce overall network stability.

## 5 Conclusion

In this study, the MNC content increased significantly following RSD treatment in the context of continuous cropping-induced soil degradation, further leading to an enhancement of the soil organic carbon pool. However, the extent of increase in MNC varied depending on the type of organic material applied under RSD. Specifically, the application of soybean straw under RSD primarily promoted the accumulation of FNC, which can be attributed to its stronger efficacy in suppressing soil-borne pathogenic fungi. This effect was also closely associated with a more pronounced proliferation of anaerobic bacteria compared to the wheat straw treatment. In contrast, the use of wheat straw primarily enhanced the accumulation of BNC, likely due to a more substantial bacterial succession relative to the soybean straw amendment. Therefore, in RSD practice, if the objective is rapid carbon sequestration and strong pathogen suppression, low C/N ratio materials (e.g., legume straw) are recommended; whereas if the goal is to foster a diverse and functionally versatile bacterial community for long-term soil health, higher C/N ratio materials (e.g., grass straw) may be preferable.

## Data Availability

The 16S rRNA and ITS gene sequences obtained in this study have been deposited in the NCBI Sequence Read Archive (SRA) database under BioProject number PRJNA1322145.

## Footnotes

1
http://www.majorbio.com
