# Supplementary material for: Bacterial functional traits: a key driver of soil organic carbon dynamics during reductive soil disinfestation
Source: Front Microbiol. 2026 Apr 22;17:1784827. doi: 10.3389/fmicb.2026.1784827 (PMC13143972; doi:10.3389/fmicb.2026.1784827)
Supplement: Supplementary file 1 [file Supplementary_file_1.docx]

**Research article**

**Title:**

**Bacterial functional traits: a key driver of soil organic carbon dynamics during reductive soil disinfestation**

Risheng Xu ^1,2†^, Haijiao Liu ^1,2,†^, Juan Liu ^1,2^, Yafei Chen ^1,2^, Ruiwei Ran ^1^, Lina Gao ^1^, Weizhen Zhao ^1^, Xing Chen ^3 *^, Tengfei Ma ^1,2 *^

^1^ Peanut Research Institute, Henan Academy of Agricultural Sciences, Zhengzhou 450002, China;

^2^ Key Laboratory of Oil Crops in Huanghuaihai Plains, Ministry of Agriculture and Rural Affairs /Henan Provincial Key Laboratory for Oil Crops Improvement，Zhengzhou 450002, Henan, China

^3^ Sanming University, San’ming 365004, China;

^*^Correspondence: chenxing162@mail.ucas.ac.cn (X.C.); matengfei@hnagri.org.cn (T.M.)

**Author for correspondence: Xing Chen**

Email: jpshen@fjnu.edu.cn

Sanming University, San’ming 365004, China

**Author for correspondence: Tengfei Ma**

Email: matengfei@hnagri.org.cn

Peanut Research Institute, Henan Academy of Agricultural Sciences, Zhengzhou 450002, China

^†^These authors contributed equally to this work.

**Supplementary methods**

***Phospholipid fatty acid (PLFA) analys*** The living microbial community structure was assessed via phospholipid fatty acid (PLFA) analysis. Lipids were extracted from 8 g of freeze-dried soil using a phosphate buffer-chloroform-methanol mixture (0.8:1:2, v/v/v). Phospholipids were methylated to form fatty acid methyl esters, which were quantified using an Agilent 7890B gas chromatograph (Agilent Technologies, Santa Clara, CA, USA). Peaks were identified with the Microbial Identification System (MIDI Inc., Newark, DE, USA).

Individual PLFA peaks were identified with a Microbial Identification System (MIDI Inc., Newark, DE, USA). PLFA markers used to represent different microbial groups—including fungi, saprotrophic fungi (SF), arbuscular mycorrhizal fungi (AM), total bacteria, aerobes, anaerobes, gram-positive, and gram-negative bacteria—are summarized in Table 1.

**Supplementary table 1** Selected PLFA markers used to characterize the soil microbial community

| Microorganisms | Markers of PLFAs | References |
| --- | --- | --- |
| Fungi | 18:1w9c | Rousk et al., 2007 |
| Arbuscular mycorrhiza | 16:1ω5c | Olsson et al., 1999 |
| Saprophytic fungi | 18:2ω6, 18:1ω9 | Olsson et al., 1999 |
| Bacteria | 11:0; 12:0; 13:0; 14:0; 15:0; 16:1; 17:0; 18:1; 19:0; i15:0; a15:0; i16:0; a17:0; i17:0; 16:1ω7c; 18:1ω7c | Rousk et al., 2007 |
| Anaerobic bacteria | i-15:0; a-15:0; a-17:0; i-17:0; a-17:0 | Moche et al., 2015 |
| Aerobic bacteria | 14:1; 18:1ω7c; 15:1ω6c; 16:1ω7c; 16:1ω7t; 18:1ω9c; 18:1ω9t | Moche et al., 2015 |
| Gram-negative bacteria | i15:0; a15:0; i16:0; 16:1w9c; 16:1w7; i17:0; a17:0; 18:0; 18:1w9c | Marshall et al., 2011 |
| Gram-positive bacteria | i16:1w7c; i17:1w8c; cy17:0; 18:1w7c; 18:1w5c; cy19:0 | Marshall et al., 2011 |

***PCR amplification*** Each 50 μL PCR reaction solution consisted of 2 μL template DNA (10 ng), 1 μL (10 μM) of each primer, 0.4 µL FastPfu Polymerase, 2 µL 2.5 mM dNTPs, 4 µL 5 × FastPfu Buffer, and ddH_2_O. The PCR reaction was conducted in triplicate on a MyCycler Thermal Cycler (Bio-Rad), and the procedure was as follows: initial denaturation at 94 °C for 2 min, followed by 25 cycles at 94 °C for 30 s, 55 °C for 30 s, and 72 °C for 45 s, with final elongation at 72 °C for 10 min. The PCR products from each sample were mixed and purified using a DNA gel extraction kit (Takara, Dalian, China).

***Sequence data analysis*** Raw data from high-throughput sequencing were demultiplexed and mass filtered. Sequences were discarded if they had one mismatch with the barcode sequence, had more than two mismatches with the primers, contained any ambiguous bases, had an average quality score of < 30, or had a minimum sequence length of < 200 bp. Denoised reads were dereplicated and processed using the DADA2 plugin for QIIME2 (Callahan et al., 2016). Amplicon sequence variants (ASVs) with read counts were generated using the default DADA2 pipeline with a 97% identity. After removing singleton ASVs, bacterial and fungal taxonomy was classified based on a confidence threshold of 70% in the Silva (Release 132) and UNITE (Release 7.2) databases, respectively. The conservative threshold for ASV filtration was 0.005%. After the contaminating mitochondrial and chloroplast sequences were removed, the alpha diversity indices (such as sobs, shannon, invsimpson, and shannon evenness) were calculated according to the ASV tables. The rrnDB database was employed to assign 16S rRNA rrn copy numbers for each OTU by matching them to their closest phylogenetic relatives with documented rrn copy numbers (Stoddard et al., 2014). The annotation success rates for OTUs in our dataset across taxonomic ranks were as follows: genus (g): 54.92%; family (f): 49.32%; order (o): 56.09%; class (c): 79.64%; phylum (p): 89.20%. To maximize the estimation of rrn copy numbers, we used the lowest reliable confidence threshold of 0.1 to output the most closely related species annotation for each OTU, thereby providing a plausible upper bound for rrn abundance based on genomic potential.

***Network analysis*** Network analysis was conducted using the CoNet plugin in Cytoscape (version 3.6.1) to identify co-occurrences of bacteria and fungi (Faust et al., 2012). Data were filtered before network analysis to remove zero values arising from spurious correlations. The correlation scores (Pearson correlation, Spearman correlation, Bray-Curtis dissimilarity, Kullback-Leibler dissimilarity, and mutual information) were calculated and used to identify all the pair-wise associations. The resultant distribution was refined with 1000 bootstraps, and the ReBoot procedure was performed with 100 permutations to avoid potential compositionality biases and false-positive correlations. The Brown method was used to integrate the *p* values of the five methods, and only correlations with *p* < 0.05 were retained for the next analysis. The multiple-test hypothesis correction procedure was performed using Benjamini-Hochberg multiple tests to limit the false discovery rate. The resulting correlations were visualized using the Gephi platform (version 0.9.2; <https://gephi.org/>), and the topology property parameters were also calculated and visualized using ImageGP (https://www.bic.ac.cn/BIC/) (Chen et al., 2022).

The structural robustness (i.e., natural connectivity) of each network was calculated to compare the stability of the networks. It is an average eigenvalue derived from network spectrum, which describes the redundancy of alternative paths. A higher robustness indicates a more stable network structure.

***Function predication*** PICRUSt2 (Douglas et al., 2020) and the FUNGuild (v1.1) annotation tool (Nguyen et al. 2016) were used to predict the function of the 16S rRNA genes (the enzymes in the KEGG pathway for pectin, esterase, cellulase, and xylanase were analyzed) and assign fungal functional guilds (mostly symbiotrophs, pathotrophs, and saprotrophs).

**References**

Callahan, B.J., McMurdie, P.J., Rosen, M.J., Han, A.W., Johnson, A.J.A., Holmes, S.P., 2016. DADA2: high-resolution sample inference from Illumina amplicon data. Nature Methods 13, 581–583.

Chen, T., Liu, Y. X., Huang, L., 2022. ImageGP: An easy‐to‐use data visualization web server for scientific researchers. iMeta 1, e5.

Douglas, G.M., Maffei, V.J., Zaneveld, J.R., Yurgel, S.N., Brown, J.R., Taylor, C.M., 2020. PICRUSt2 for prediction of metagenome functions. Nature Biotechnology 38, 685–688.

Faust, K., Sathirapongsasuti, J. F., Izard, J., Segata, N., Huttenhower, C., 2012. Microbial co-occurrence relationships in the human microbiome. PLoS Computational Biology 8, e1002606.

Marshall, C.B.; McLaren, J.R.; Turkington, R., 2011. Soil microbial communities resistant to changes in plant functional group composition. Soil Biology and Biochemistry 43, 78–85.

Moche, M.; Gutknecht, J.; Schulz, E.; Langer, U.; Rinklebe, J., 2015. Monthly dynamics of microbial community structure and their controlling factors in three floodplain soils. Soil Biology and Biochemistry 90, 169–178.

Nguyen, N.H., Song, Z., Bates, S.T., Branco, S., Tedersoo, L., Menke, J., Kennedy, P.G., 2016. FUNGuild: an open annotation tool for parsing fungal community datasets by ecological guild. Fungal Ecology 20, 241–248.

Stoddard, S.F., Smith, B.J., Hein, R., Roller, B.R., Schmidt, T.M., 2014. rrnDB: improved tools for interpreting rRNA gene abundance in bacteria and archaea and a new foundation for future development. Nucleic Acids Research 43, 593–598.

Rousk, J., Bååth, E., 2007. Fungal and bacterial growth in soil with plant materials of different C/N ratios. FEMS microbiology ecology 62, 258–267.

Olsson, P.A., 1999. Signature fatty acids provide tools for determination of the distribution and interactions of mycorrhizal fungi in soil. FEMS microbiology ecology 29, 303–310.

**Supplement Figures**

**Supplementary Fig. 1.** Effects of anaerobic soil disinfestation on MNC/SOC. The different letters indicate significant differences between the treatments (*p* < 0.05). Control soil (CK); Water flooding without straw (WF); Water flooding with low-dose (1.5 g kg^-1^) soybean straw (LSD); Water flooding with high-dose (7 g kg^-1^) soybean straw (HSD); Water flooding with low-dose (1.5 g kg^-1^) wheat straw (LWD); Water flooding with high-dose (7 g kg^-1^) wheat straw (HWD).

**Supplementary Fig. 2.** Concentrations (µg/g) of bacterial, anaerobic bacteria, gram-positive and -negative bacteria, fungal PLFAs under RSD with soybean straw and wheat straw. Control soil (CK); Water flooding without straw (WF); Water flooding with low-dose (1.5 g kg^-1^) soybean straw (LSD); Water flooding with high-dose (7 g kg^-1^) soybean straw (HSD); Water flooding with low-dose (1.5 g kg^-1^) wheat straw (LWD); Water flooding with high-dose (7 g kg^-1^) wheat straw (HWD). The different letters indicate significant differences between the treatments (*p* < 0.05).

**Supplementary Fig. 3.** The analysis of similarity (ANOSIM) of microbial community structure among the treatments.

**Supplementary Fig. 4.** Absolute abundance of plant pathogen. The different letters indicate significant differences between the treatments (*p* < 0.05).

**Supplementary Fig. 1**

**
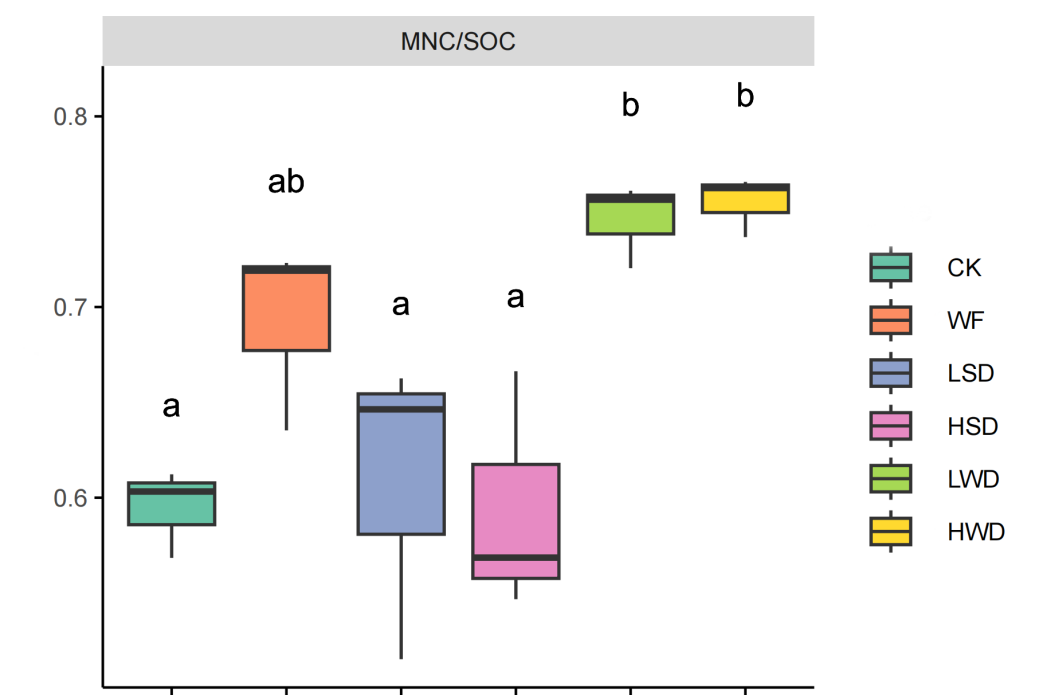
**

**Supplementary Fig.2**


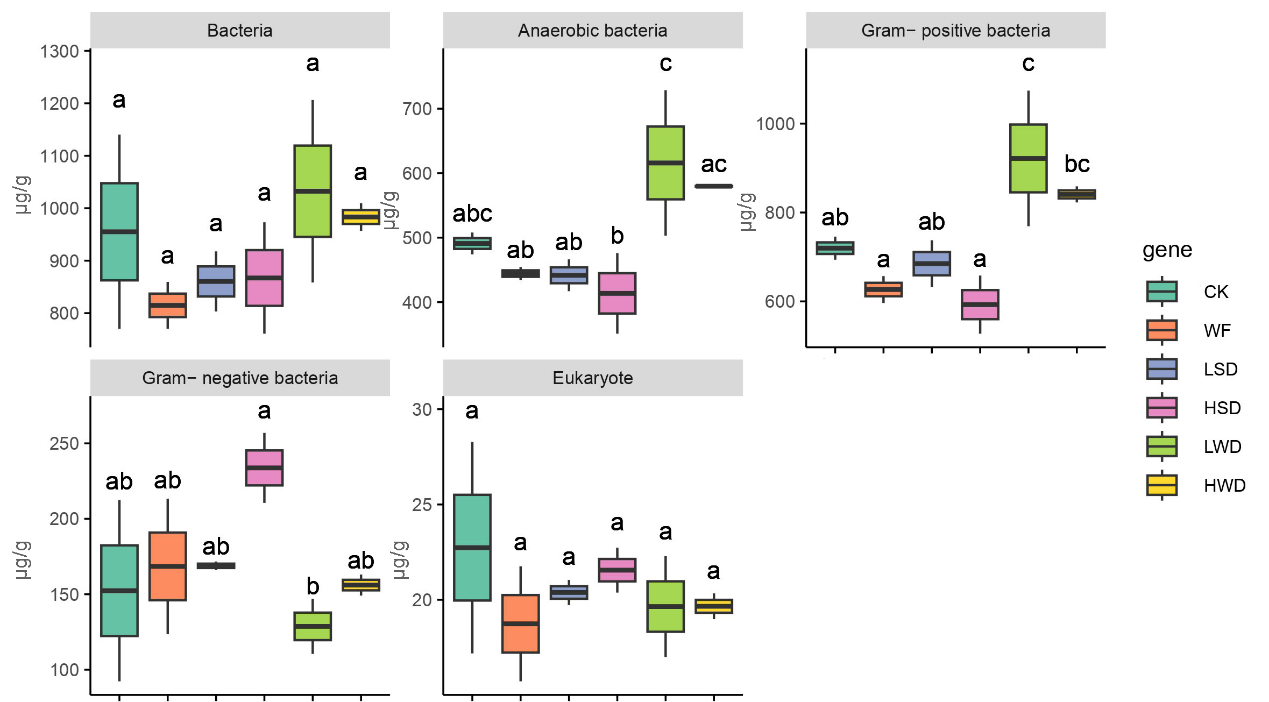


**Supplementary Fig. 3**


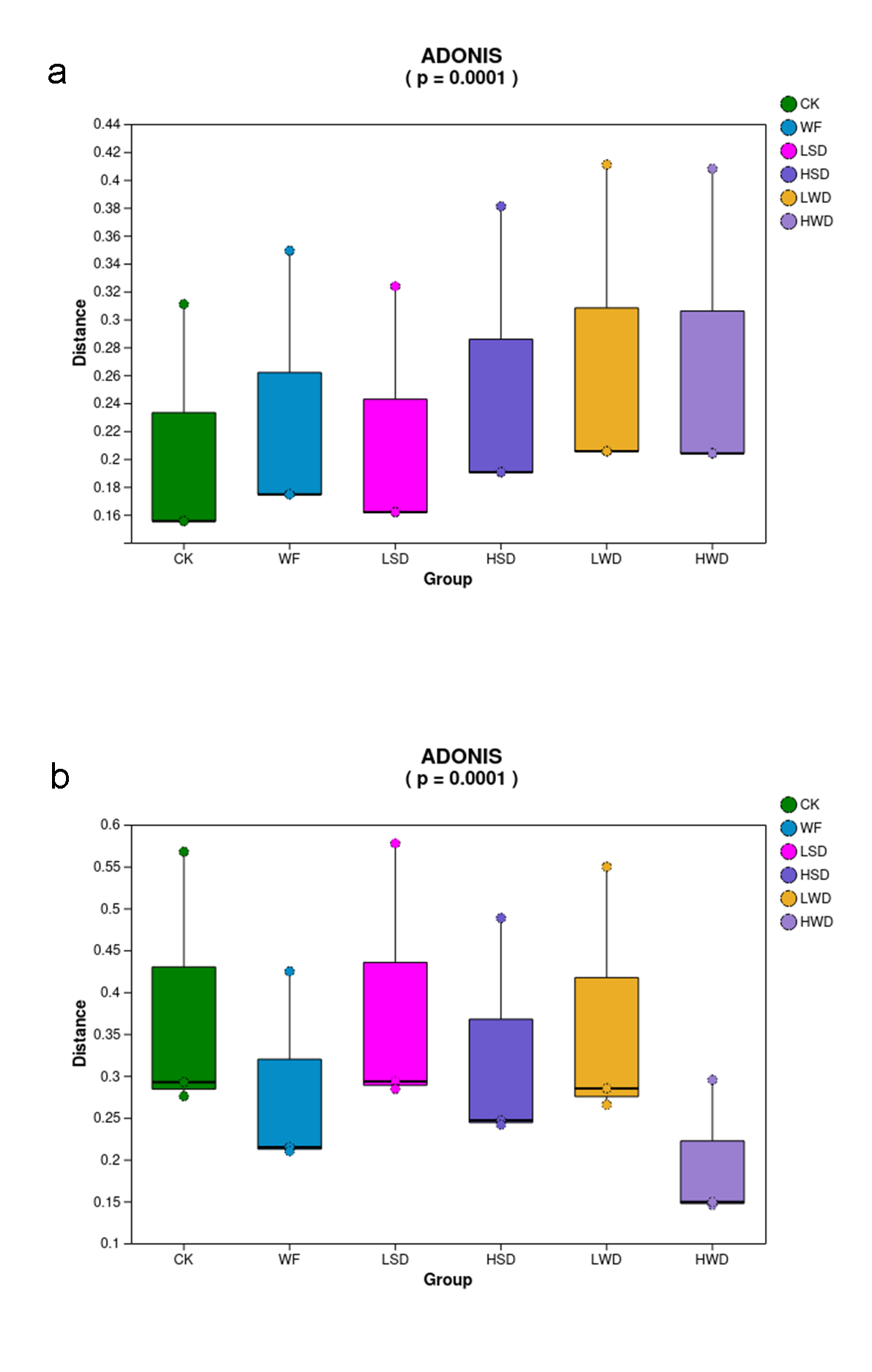


**Supplementary Fig. 4**


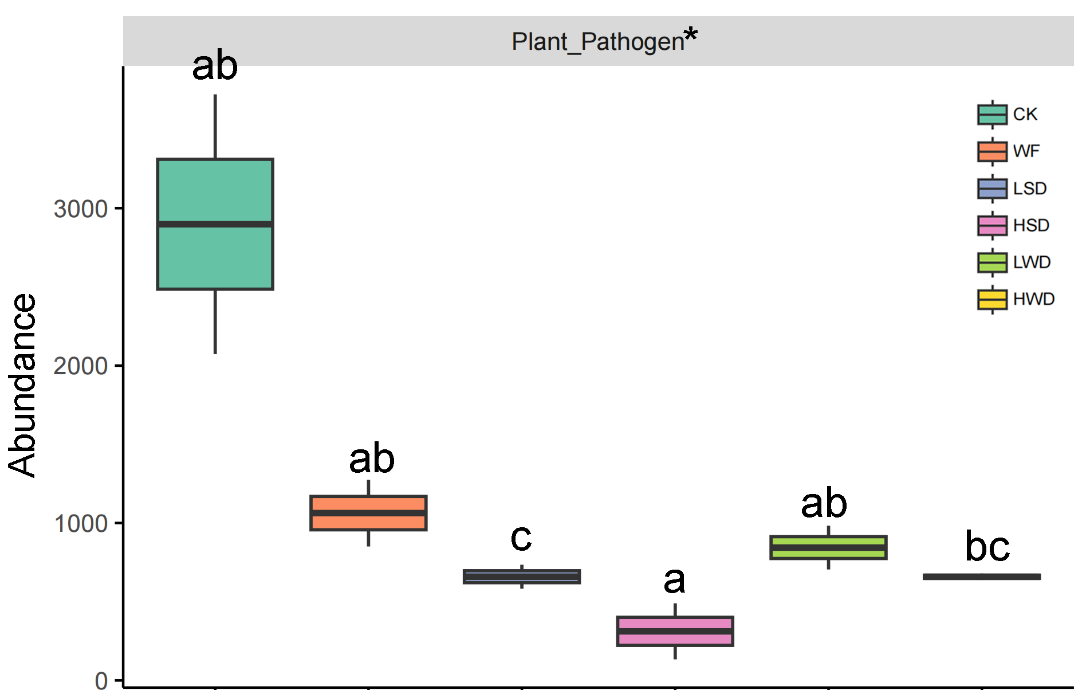


**Supplementary table 2** Chemical properties of soil acorss different treatments.

|  | CK | WF | LSD | HSD | LWD | HWD |
| --- | --- | --- | --- | --- | --- | --- |
| pH | 8.5±0.0e | 8.3±0.0d | 8.0±0.0b | 7.7±0.1a | 8.1±0.1c | 7.8±0.0a |
| Eh (mV)* | 505±8.3e | -58±1.5d | -84.3±4.5ac | -103.3±3.0b | -68.7±8.3ed | -94.7±5.8ab |
| EC (us cm^-1^)* | 507±7.9e | 449±7.8d | 175.7±11.5ab | 150±9.5b | 237.3±14.8ac | 193.3±19.8a |
| NO_3_^-^ (mg kg^-1^) | 218.7±7.5b | 132.7±6.6a | 126±3.6a | 95.7±4.9c | 133.3±4.4a | 125.3±6.3a |
| NH_4_^+^ (mg kg^-1^) | 1.8±0.0d | 2.4±0.2a | 2.8±0.0bc | 3.0±0.1c | 2.6±0.1ab | 2.8±0.1bc |
| AP (mg kg^-1^) | 55.3±1.5b | 56.7±1.2ab | 61.7±0.7c | 65±1.5d | 59.7±0.9ac | 65.7±0.9d |

Values are means ± standard error (n = 3). Different lowercase letters indicate significant differences at *p* < 0.05. Eh: oxidation-reduction potential; EC: electrical conductivity; NO_3_^-^ : nitrate ion; NH_4_^+^: ammonium ion; AP: available phosphorous.

**Supplementary table 3** Effect of different organic matter addition on soil enzyme activities.

|  | CK | WF | LSD | HSD | LWD | HWD |
| --- | --- | --- | --- | --- | --- | --- |
| β-1,4-glucosidase activity(nmol g^-1^ h^-1^) | 1.82±0.05d | 1.99±0.02cd | 2.19±0.06bc | 2.49±0.07a | 2.02±0.08cd | 2.28±0.09b |
| Cellobiohydrolase activity(nmol g^-1^ h^-1^) | 0.46±0.03f | 0.57±0.03e | 0.94±0.05c | 1.26±0.03a | 0.75±0.03d | 1.08±0.02b |
| β-1,4-xylosidase activity(nmol g^-1^ h^-1^) | 0.12±0.02e | 0.22±0.02d | 0.37±0.03bc | 0.56±0.04a | 0.32±0.02c | 0.42±0.04b |

Values are means ± standard error (n = 3). Different lowercase letters indicate significant differences at *p* < 0.05.

**Supplementary table 4** α-diversity of bacterial communities across all treatments

|  | CK | WF | LSD | HSD | LWD | HWD |
| --- | --- | --- | --- | --- | --- | --- |
| Chao1 | 1394.44±99.46b | 1495.20±157.08b | 1793.02±113.12b | 1329.79±90.10b | 1493.48±51.58b | 2514.06±235.57a |
| Dominance | 0.0045±0.0003b | 0.0040±0.00058b | 0.0035±0.0003b | 0.0125±0.0026a | 0.0050±0.0006b | 0.0035±0.0003b |
| Goods coverage* | 0.9985±0.0003a | 0.9980±0.0006a | 0.9965±0.0003a | 0.9975±0.0009b | 0.9985±0.0003a | 0.9925±0.0014a |
| Observed features | 1385.00±96.42bc | 1482.00±154.73bc | 1738.00±113.16b | 1300.50±77.08c | 1478.50±52.83bc | 2406.00±203.80a |
| Pielou e | 0.8675±0.0003b | 0.876±0.0058b | 0.8765±0.0032b | 0.8045±0.0124a | 0.8735±0.0101b | 0.8740±0.0023b |
| Shannon | 9.0420±0.0849c | 9.2045±0.1943bc | 9.4230±0.0485b | 8.3120±0.0589d | 9.1970±0.0606bc | 9.8030±0.0808a |
| Simpson | 0.9955±0.0003b | 0.9960±0.0006b | 0.9965±0.0003b | 0.9875±0.003a | 0.9950±0.0006b | 0.9965±0.0003b |

Values are means ± standard error (n = 3). Different lowercase letters indicate significant differences at *p* < 0.05.

**Supplementary table 5** α-diversity of fungal communities across all treatments

|  | CK | WF | LSD | HSD | LWD | HWD |
| --- | --- | --- | --- | --- | --- | --- |
| Chao1 | 857.94±51.23b | 1567.19±256.81a | 1085.93±9.15b | 844.72±37.21b | 1001.53±53.40b | 1039.79±0.16b |
| Dominance* | 0.0195±0.0020b | 0.0200±0.0023b | 0.1280±0.0052a | 0.0600±0.0040b | 0.0210±0.0012a | 0.0710±0.0173a |
| Goods *coverage | 1.0000±0.0000a | 0.9995±0.0003ab | 0.9990±0.0000b | 0.9995±0.0003ab | 1.0000±0.0000a | 1.0000±0.0000a |
| Observed features | 855.50±51.10b | 1550.50±250.86a | 1067.50±6.64b | 836.00±35.22b | 994.00±51.96b | 1032.50±0.29b |
| Pielou e* | 0.7790±0.0052a | 0.7440±0.0023a | 0.5190±0.0058c | 0.5955±0.0142bc | 0.7555±0.0078a | 0.6375±0.0332b |
| Shannon* | 7.5855±0.1187a | 7.8425±0.2053a | 5.2210±0.0652c | 5.7760±0.1028b | 7.5140±0.0208a | 6.3860±0.3314b |
| Simpson* | 0.9805±0.0020a | 0.9800±0.0023a | 0.8720±0.0052c | 0.9400±0.0040b | 0.9790±0.0012a | 0.9290±0.0173b |

Values are means ± standard error (n = 3). Different lowercase letters indicate significant differences at *p* < 0.05.

**Supplementary table 6.** Topological properties of soil bacteria community networks

|  | **CK** | **SD** | **WD** |
| --- | --- | --- | --- |
| Modularity | 0.879 | 0.88 | 0.872 |
| Degree | 46.313 | 50.850 | 48.593 |
| Density | 0.039 | 0.043 | 0.034 |
